# A study on prospective associations between adiposity and 7-year changes in movement behaviors among older women based on compositional data analysis

**DOI:** 10.1186/s12877-021-02148-3

**Published:** 2021-03-23

**Authors:** Jana Pelclová, Nikola Štefelová, Timothy Olds, Dorothea Dumuid, Karel Hron, Sebastien Chastin, Željko Pedišić

**Affiliations:** 1 Faculty of Physical Culture, Palacký University Olomouc, Institute of Active Lifestyle, Tř. Míru 117, 771 11 Olomouc, Czech Republic; 2 Faculty of Science, Palacký University Olomouc, Olomouc, Czech Republic; 3 Alliance for Research in Exercise, Nutrition and Activity (ARENA), University of South Australia, Adelaide, South Australia Australia; 4 School of Health and Life Science, Glasgow Caledonian University, Glasgow, UK; 5 Institute for Health and Sport, Victoria University, Melbourne, Australia

**Keywords:** Compositional data analysis, Time-use epidemiology, Fatness, Obesity, Sitting, Exercise

## Abstract

**Introduction:**

It is unclear whether adiposity leads to changes in movement behaviors, and there is a lack of compositional analyses of longitudinal data which focus on these associations. Using a compositional approach, this study aimed to examine the associations between baseline adiposity and 7-year changes in physical activity (PA) and sedentary behavior (SB) among elderly women. We also explored the longitudinal associations between change in adiposity and change in movement-behavior composition.

**Methods:**

This longitudinal study included 176 older women (mean baseline age 62.8 (4.1) years) from Central Europe. Movement behavior was assessed by accelerometers and adiposity was measured by bioelectrical impedance analysis at baseline and follow-up. A set of multivariate least-squares regression analyses was used to examine the associations of baseline adiposity and longitudinal changes in adiposity as explanatory variables with longitudinal changes in a 3-part movement-behavior composition consisting of SB, light PA (LPA) and moderate-to-vigorous PA (MVPA) as outcome variables.

**Results:**

No significant associations were found between baseline adiposity and longitudinal changes in the movement-behavior composition (*p* > 0.05). We found significant associations of changes in body mass index (BMI) and fat mass percentage (FM%) with changes in the movement-behavior composition. An increase in BMI was associated with an increase of SB at the expense of LPA and MVPA (*β* = 0.042, *p* = 0.009) and with a decrease of MVPA in favor of SB and LPA (*β* = − 0.059, *p* = 0.037). An increase in FM% was significantly associated only with an increase of SB at the expense of LPA and MVPA (*β* = 0.019, *p* = 0.031).

**Conclusions:**

This study did not support the assumption that baseline adiposity is associated with longitudinal changes in movement behaviors among elderly women, but we found evidence for change-to-change associations, suggesting that a 7-year increase in adiposity is associated with a concurrent increase of SB at the expense of LPA and MVPA and with a concurrent decrease of MVPA in favor of LPA and SB. Public health interventions are needed to simultaneously prevent weight gain and promote physically active lifestyle among elderly women.

**Supplementary Information:**

The online version contains supplementary material available at 10.1186/s12877-021-02148-3.

## Introduction

Previous studies have found that physical activity (PA) and sedentary behavior (SB) are associated with adiposity [1–4]. In prospective cohort studies, low PA and high SB at baseline have usually been associated with higher subsequent adiposity [5, 6]. However, some studies provided evidence suggesting that adiposity may lead to subsequent changes in movement behaviors [7]; specifically, to reduced PA [8] and increased SB [9]. It seems, therefore, that the association between adiposity and movement behaviors may be bidirectional.

Higher PA may plausibly lead to lower adiposity through higher energy expenditure, but conversely higher adiposity may lead to reductions in PA through increased discomfort and lower fitness. The same applies mutatis mutandis to SB.

Importantly, PA and SB are co-dependent. Since there are only 24 h in a day, any change to the amount of time we spend in one of the two behaviors must be compensated by an equal and opposite change in the other behavior and sleep collectively [10, 11]. It therefore makes more sense to talk about activity compositions rather than individual movement behaviors, and to ask whether composition of activity is related to adiposity. For this purpose, it is recommended to use compositional data analysis [10–14]. Some studies have found significant cross-sectional relationships, with compositions with high levels of moderate to vigorous PA (MVPA) being favorably associated with adiposity [10]. This relationship has also been explored in several longitudinal studies [15, 16]. However, there is a lack of compositional analyses of longitudinal data which focus on the association between baseline adiposity and subsequent changes in movement behaviors.

Using a compositional approach, this study, therefore, aimed to examine the prospective associations between baseline adiposity and 7-year changes in PA and SB among elderly women. We also explored the longitudinal associations between change in adiposity and change in movement-behavior composition.

## Methods

### Design and participants

**Fig. 1:**
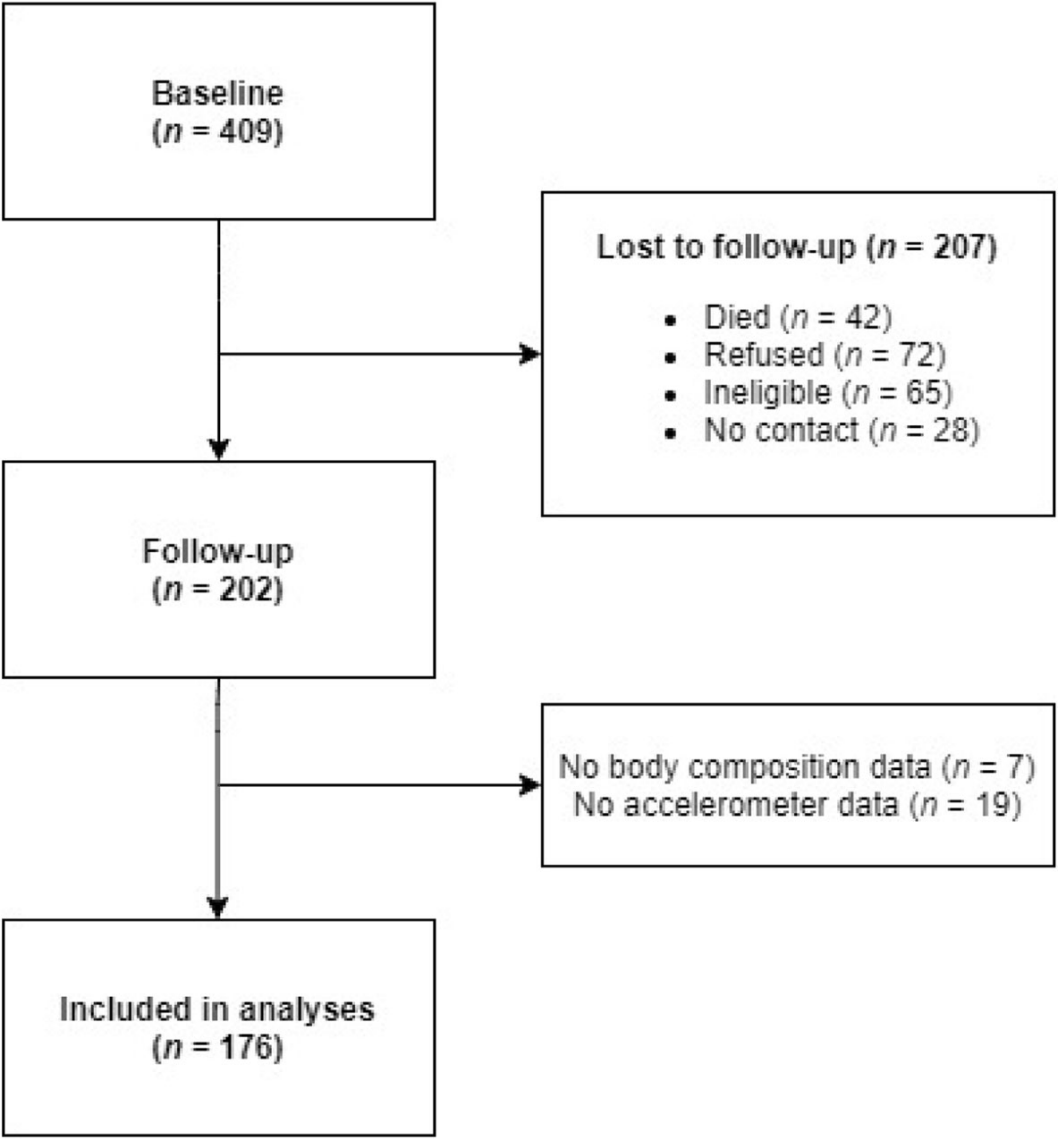
Flow diagram of participation in longitudinal study

This 7-year longitudinal study was conducted among elderly women from three Central European countries, namely, the Czech Republic, Poland and the Slovak Republic (Fig. 1). The baseline data collection was conducted between 2009 and 2012 and included 409 women aged 60+, who were able to walk without any prosthetic aids and were not living in a residential care. These women were recruited amongst attendees of University of Third Age programs. The follow-up assessments, previously scheduled by phone call, were arranged individually with each participant between 2016 and 2019. Out of the 409 baseline participants, 17.6% were not willing to participate in some part or in all parts of the follow-up assessment, 6.8% could not be contacted due to invalid phone number or address, 15.9% were unable to repeat the assessment due to short- or long-term serious health problems, and 10.3% died. All participants were informed that their participation was voluntary and that they could withdraw from the study at any time. They provided their written consent before participating in baseline and follow-up measurements. The study was approved by the Institutional Research Ethics Committee, Faculty of Physical Culture, Palacký University Olomouc. A detailed description of the study can be found elsewhere [17].

### Measures

#### Adiposity

Body Mass Index (BMI) was calculated from standing height measured using the P-375 portable stadiometer to the nearest 0.1 cm (Trystom, Olomouc, Czech Republic). Body weight was measured to the nearest 0.5 kg using the InBody 720 device (Biospace Co., Ltd., Seoul, Korea). The same device was also used to assess fat mass percentage (FM%) through bioelectrical impedance analysis. The device was found to have acceptable validity for assessing FM% [18, 19]. The participants were asked to hydrate properly for 24 h, fast for at least 2 h and avoid any vigorous activity at least 48 h before the adiposity assessment was conducted. The same protocol was followed for adiposity assessment at baseline and follow-up. For sample description, BMI was categorized as ‘normal’ weight (< 25 kg/m2), overweight (25–29.9 kg/m^2^) and obesity (≥30 kg/m^2^) [20], while body fat percentage (%BF) was classified as ‘normal’ (< 35%) and obesity (≥35%) [21].

#### Movement behaviors

To assess the amounts of time spent in PA and SB at baseline and follow-up, the participants were asked to wear an ActiGraph GT1M accelerometer (Manufacturing Technology Inc., Pensacola, FL, USA) on their right hip for at least seven consecutive days. The sampling interval was set at 1 min, and non-wear time was defined as any interval of 60 or more minutes with no activity counts, with allowance for up to 2 min with 0–100 activity counts per minute. In accordance with the study protocol [17], the participants were asked to wear the device during waking hours only, to put it on immediately after waking up and to remove it before falling asleep. They were also instructed to remove the device during water activities (e.g. swimming, bathing, showering). The participants with at least 4 days (including at least three weekdays and one weekend day) with ≥10 wear time hours were included in the data analysis. Using the ActiGraph software, the amount of time spent in SB, light PA (LPA) and moderate-to-vigorous PA (MVPA) was derived from activity counts (ActiGraph, LLC, Pensacola, FL, USA). We used the following cut-points: < 100 cpm for SB [22], 100–1951 cpm for LPA [23] and ≥ 1952 cpm for MVPA [23].

#### Other measures

Education level was self-reported by participants at baseline, and it was dichotomized as “primary/secondary education” and “tertiary degree”. Participants also self-reported their place of residence, employment status and marital status at baseline and follow-up. Employment status was categorized as “employed”, “unemployed” and “retired”. Marital status was dichotomized as “living alone” and “living with a partner”. Health status was assessed using open-ended questions on different illness or disability occurrence before baseline and during the 7-year period between baseline and follow-up. The participants provided information about occurrence of cardiovascular disease, musculoskeletal disorders, chronic respiratory diseases, cancer, diabetes, urogenital or endocrine diseases, and osteoporosis before baseline and during the 7-year follow-up period. Health status was dichotomized as “illness/disability occurrence” and “no illness/disability occurrence”.

### Statistical analysis

Statistical analyses were performed using the R Statistical Software, version 3.4.2 [24] using the compositions [25] package and the IBM Statistical Package for the Social Sciences software, version 23 (SPSS Inc., an IBM Company, Armonk, NY, USA). To examine the prospective association between baseline adiposity and 7-year changes in a 3-part movement-behavior composition consisting of SB, LPA and MVPA, we used compositional data analysis (CoDA) [26]. Compositional data analysis acknowledges the relative nature of time-use data by expressing them as a set of log-ratios. The log-ratios can then be analysed instead of raw absolute values (e.g., minutes of MVPA), using standard statistical models [27]. Movement compositions were mapped into real Euclidean space using specific isometric log-ratio coordinates called pivot coordinates [28].

We conducted a set of multivariate robust compositional regression analyses with changes in movement behaviors (calculated from pivot coordinates of baseline and follow-up movement compositions) as dependent variables and baseline BMI, (baseline FM% respectively), change in BMI (and change in FM% respectively) as explanatory variables. The regression models were adjusted for baseline age, country, follow-up duration, baseline wear-time, follow-up wear-time, baseline movement-behavior composition, change in adiposity, education, residence change (dummy variable), baseline employment status, change in employment status (dummy variables representing no change, transition to retirement, and transition from unemployment to employment), baseline health status, change in health status, baseline marital status and follow-up marital status. The necessary adjustments for confounding were determined using a directed acyclic graph (DAG), where the causal assumptions were made based on findings from previous studies among older adults [29]. Three models were run, each with a different permutation of the movement behaviors within the pivot co-ordinates, to allow interpretation of each movement behavior relative to other movement behaviors. Significance level was set at *p* < 0.05. A comprehensive explanation of the compositional analysis is included in Supplementary file.

## Results

### Sample characteristics

The formation of the final study sample is shown in Fig. 1. Out of 202 women who attended follow-up, 176 had valid data. Of these, 44.9, 35.8 and 19.3% were from the Czech Republic, Poland and the Slovak Republic, respectively. At baseline, most of the participants were retired (73.3%) and lived with a partner (73.9%). During the 7 years between baseline and follow-up, 67.6% of participants reported occurrence of disability or illness, 52.8% (out of 36 participants who were employed at baseline) retired and 5% of participants changed their place of residence. At follow-up, 41.6% lived alone.

### Longitudinal changes in adiposity and movement-behavior composition

The mean and standard deviation of follow-up duration was 84 (6) months. The mean daily accelerometer wear-time was 14.1 (1.2) and 13.6 (1.2) hours/day at baseline and follow-up, respectively. Descriptive statistics for adiposity measures and movement-behavior compositions are presented in Table 1. There were significant longitudinal changes in BMI and adiposity (Table 1). During the study period, BMI and FM% increased on average by 1 kg/m^2^and 2.3%, respectively (*p* < 0.001 for both). According to BMI, the prevalence of obesity increased from 19% at baseline to 24% at follow-up (*p* = 0.01). According to FM% the prevalence of obesity increased from 47% at baseline to 61% at follow-up (*p* = 0.01). During the 7 years, the average amount of time spent in SB increased by 14%, while the average amounts of time spent in LPA and MVPA decreased by 15 and 17.5%, respectively. The prevalence of women meeting the recommendation of 150 min/week of MVPA [30–32] decreased from 78.2% at baseline to 64.3% at follow-up. Furthermore, the prevalence of women sitting less than 8 h a day decreased from 70.5% at baseline to 52.3% at follow-up.

**Table 1:** Sample characteristics at baseline and follow-up

	Baseline		Follow-up		Difference^**a**^		
	Mean	Var	Mean	Var	Mean	Var	***p***
Age (years)	62.8	4.1	69.8	4.2	7.0	0.6	< 0.001
Height (cm)	160.8	6.5	159.7	6.7	−1.0	1.4	< 0.001
Weight (kg)	68.3	10.3	69.7	11.2	1.4	4.8	< 0.001
BMI (kg/m^2^)	26.4	4.0	27.3	4.2	0.9	1.9	< 0.001
**Body composition**							
Fat mass percentage (%)	34.6	6.3	36.8	6.6	2.2	3.8	< 0.001
**Movement-behavior composition**							
SB (%)	52.2	58.9	59.5	59.0	7.3		
LPA (%)	43.8	51.9	37.2	49.4	−6.6		< 0.001
MVPA (%)	4.0	89.3	3.3	91.6	−0.7		

Mean = Arithmetic mean for non-compositional variables and compositional mean for time-use components expressed in percentages, Var = Standard deviation for non-compositional variables and the percentage of total variance of log-ratios related to a given time-use component, *p* = *p*-value from the paired t-test for non-compositional variables and Hotelling test for movement behavior composition), BMI = body mass index, SB = time spent in sedentary behavior, LPA = time spent in light physical activity, MVPA = time spent in moderate-to-vigorous physical activity

^a^ difference in movement composition variables was expresses as follow-up % minus baseline %

### Association between adiposity and 7-year changes in movement-behavior composition

We did not find significant associations of baseline BMI and FM% with longitudinal changes in movement-behavior composition (Table 2). We found significant associations between changes in adiposity from baseline to follow-up and changes in some parts of the movement-behavior composition from baseline to follow-up (Table 2). Specifically, an increase in BMI was associated with an increase of SB at the expense of LPA and MVPA (*β* = 0.042, *p* = 0.009) and with an increase of SB at the expense of MVPA (*β* = 0.057, *p* = 0.021). An increase in BMI was also associated with a decrease of MVPA in favor of SB and LPA (*β* = − 0.059, *p* = 0.037). An increase in FM% was significantly associated only with an increase of SB at the expense of LPA and MVPA (*β* = 0.019, *p* = 0.031). We did not find any significant associations between BMI or FM% change and change in LPA (relative to the remaining movement behaviors).

**Table 2:** Associations between adiposity and 7-year changes in movement-behavior composition: results of a least-squares regression analysis

	Model 1				Model 2				Model 3			
	(SB/LPA + SB/MVPA) difference		(LPA/MVPA) difference		(LPA/SB + LPA/MVPA) difference		(SB/MVPA) difference	(MVPA/SB + MVPA/LPA) difference				(SB/LPA) difference
	*β* (95% CI)	*p*	*β* (95% CI)	*p*	*β* (95% CI)	*p*	*β* (95% CI)	*p*	*β* (95% CI)	*p*	*β* (95% CI)	*p*
Baseline BMI (kg/m^2^)	0.002 (− 0.014, 0.018)	0.848	0.009 (− 0.013, 0.031)	0.434	0.010 (− 0.013, 0.033)	0.40	0.009 (− 0.011, 0.029)	0.382	−0.011 (− 0.033, 0.012)	0.342	− 0.004 (− 0.017, 0.010)	0.604
Change in BMI (kg/m^2^)	0.042 (0.011, 0.073)	0.009	0.045 (−0.008, 0.098)	0.095	0.02 (−0.036, 0.075)	0.487	0.057 (0.009, 0.105)	0.021	−0.059 (− 0.115, − 0.004)	0.037	0.026 (− 0.006, 0.058)	0.115
Baseline FM%	0.007 (− 0.003, 0.016)	0.150	0.013 (− 0.001, 0.027)	0.072	0.009 (− 0.002, 0.019)	0.126	0.013 (− 0.001, 0.026)	0.072	− 0.015 (− 0.031, 0.000)	0.056	0.001 (− 0.005, 0.008)	0.679
Change in FM%	0.019 (0.008, 0.036)	0.031	0.020 (−0.004, 0.045)	0.108	0.009 (−0.010, 0.029)	0.351	0.025 (−0.001, 0.052)	0.057	−0.027 (− 0.055, 0.001)	0.062	0.011 (− 0.001, 0.022)	0.082

*Note:* All models were adjusted for age, country, education, follow-up duration, baseline and follow-up wear time, baseline movement-behavior composition, residence change, baseline employment status, change in employment status, baseline health status and change in health status, baseline marital status and follow-up marital status. Baseline adiposity models were additionally adjusted for change in adiposity, while change in adiposity models were additionally adjusted for baseline adiposity. The outcome variable in each model represents the difference between the respective pivot coordinates at follow-up and baseline. For ease of interpretation, the first pivot coordinate has been expressed as the sum of individual log-ratios

A positive beta suggests that an increase in the respective explanatory variable is associated with an expected increase in the time spent in the movement behavior that is in the numerator of the pivot coordinate at the expense of the movement behavior(s) that is (are) in the denominator of the pivot coordinate. A negative beta suggests that an increase in the respective explanatory variable is associated with an expected decrease in the time spent in the movement behavior that is in the numerator of the pivot coordinate in favor of the movement behavior(s) that is (are) in the denominator of the pivot coordinate

*β* = unstandardised regression coefficient, expected change in the pivot coordinate associated with a unit increase in the explanatory variable, *CI* confidence interval, *SB* sedentary behavior, *LPA* light physical activity, *MVPA* moderate-to-vigorous physical activity, *BMI* body mass index, *FM%* fat mass percentage, *Change* change baseline to follow-up

## Discussion

Our results suggest that among elderly women a longitudinal change in adiposity over 7 years is associated with a concurrent change in movement-behavior composition. We did not find a significant prospective association between baseline adiposity and 7-year changes in movement-behavior composition.

Longitudinal changes in adiposity and movement-behavior composition found in our study concur with previous findings of age-related increase in adiposity [5, 6] and time spent in SB [33–35] and age-related decrease PA [36–38]. Furthermore, over the 7-year study period, the elderly women in our sample became more sedentary and less physically active. The increase of SB at the expense of MVPA is broadly considered to increase the risk of adverse health outcomes [39, 40].

The results of this study did not provide clear evidence of the association between baseline adiposity and subsequent changes in movement-behavior composition. Some previous prospective studies suggest obesity might be a risk factor for decrease in PA [8] or may lead to increase in SB [9]. However, these studies included mainly middle-aged populations and did not consider the movement behaviors as compositional data. Given the lack of previous evidence for prospective associations between adiposity and subsequent changes in movement-behavior composition, it is not possible to make direct comparisons of our results with findings from previous studies. Some of the non-significant associations between baseline adiposity and subsequent changes in movement behaviors found in our study sample point in the same direction as the associations found in previous, non-CoDA based studies [8, 9, 41]. For example, in our sample, higher baseline FM% was associated with an increase of SB at the expense of MVPA and with a decrease of MVPA in favor of SB and LPA. It is possible that these associations were not significant, because the sample in our study was smaller than in the two previous studies [9, 41]. A post hoc analysis of statistical power revealed that our sample size was large enough to ensure > 0.99 probability of obtaining a significant beta coefficient, if the true effect size in the population is at least medium (*f*^2^ ≥ 0.15) [42]. The probability of obtaining a significant beta coefficient in case the true effect size in the population is small (*f*^2^ ≥ 0.02) was 0.46, which is relatively low. However, Pulsford and colleagues [41] did not find a prospective association between baseline adiposity and total sitting time either, despite the fact their study was conducted in a much larger sample. The lack of significant associations in our study and in the Pulsford et al. study [41] might therefore also be an indication of a very low or no prospective association between adiposity and movement behaviors. Given the inconsistencies in findings between studies, this should be further investigated.

Although there were no prospective associations, we found significant longitudinal change-to-change associations. Our findings suggested that 7-year increases in both BMI and FM% were associated with a concurrent increase of SB at the expense of other movement behaviors. And conversely, longitudinal decrease in BMI was associated with a decrease of MVPA in favor of other movement behaviors. It is possible that the change in adiposity is more important in determining concurrent changes to movement behaviors than starting levels of adiposity. This may reflect older women’s decreasing ability to participate in MVPA and increasing propensity for SB and LPA as their adiposity increases. However, given that these were change-to-change associations, we are unable to determine the direction of this association and more research is needed to confirm these assumptions.

The main strengths of this study are its longitudinal design, the use of CoDA, and the assessment of movement behaviors using accelerometers. Additionally, the carefully selected adjustments for confounding strengthen the findings of this study. This study was subject to several limitations. The sample cannot be considered as fully representative of the population of elderly women in the Czech Republic, Poland and the Slovak Republic. The findings should, therefore, be generalized with caution. Furthermore, we did not find significant differences in baseline movement behaviours between the participants included in the analysis and those who were lost to follow-up. However, the latter group had a significantly higher age (by on average 2.03 years), BMI (by on average 1.1 kg/m^2^), and fat mass percentage (by on average 2.3%) compared with those who remained in the study. This may also have reduced the generalisability of findings. There are also limitations related to the assessment of movement behaviors. We did not measure sleep duration, despite the fact that sleep has been found to be associated with adiposity [43] and is co-dependent with SB, LPA and MVPA. A potential limitation may stem from our choice of the threshold for non-wear time. The large amount of time that older adults spend in sedentary behaviours, increases the likelihood of misclassifying sedentary time as non-wear time [44]. Some authors have, therefore, suggested using longer thresholds for non-wear time for older adults [45]. Despite that, the 60-min threshold that we used in the current paper remains the most commonly used threshold in epidemiological studies conducted in this age group [22]. Furthermore, the accelerometer cut-points used to determine the time spent in movement behaviors were validated in samples with somewhat different characteristics compared with our study sample. We also did not consider other characteristics movement behaviors such as bout length, timing and consistency, some of which have been associated with adiposity in older women [15]. Lastly, although the regression models were adjusted for illness or disability occurrence before baseline and during the 7-year follow-up, it may be that some aspects of illness and disability were not fully captured by this question. This may have led to residual confounding.

## Conclusion

This longitudinal study with device-measured daily composition of movement behaviors did not support the assumption that baseline adiposity is associated with longitudinal changes in movement behaviors among elderly women. However, we found evidence for change-to-change associations, suggesting that a 7-year increase in adiposity is associated with a concurrent increase of SB at the expense of LPA and MVPA and with a concurrent decrease of MVPA in favor of LPA and SB. Public health interventions are needed to simultaneously prevent weight gain and promote physically active lifestyle in the population of elderly women.

## Supplementary Information

**Additional file 1: Supplementary material**: Formulas of multiple regression models.

## Data Availability

The datasets generated during and/or analyzed during the current study are available in the Figshare repository, 10.6084/m9.figshare.13208000.v2
