# Supplementary material for: A study on prospective associations between adiposity and 7-year changes in movement behaviors among older women based on compositional data analysis
Source: BMC Geriatr. 2021 Mar 23;21:203. doi: 10.1186/s12877-021-02148-3 (PMC7988941; doi:10.1186/s12877-021-02148-3)
Supplement: Supplementary file 1 — Additional file 1: Supplementary material: Formulas of multiple regression models. [file 12877_2021_2148_MOESM1_ESM.pdf]

Supplementary material: Formulas of multiple regression models

A study on prospective associations between adiposity and 7-year changes in movement behaviors among older women based on compositional data analysis

Jana Pelclová[1*], Nikola Štefelová[2], Timothy Olds[3], Dorothea Dumuid[3], Karel Hron[2], Sebastien Chastin[4], Željko Pedišić[5]

[1] Faculty of Physical Culture, Palacký University Olomouc, Olomouc, Czech Republic

[2] Faculty of Science, Palacký University Olomouc, Olomouc, Czech Republic

[3] Alliance for Research in Exercise, Nutrition and Activity (ARENA), University of South Australia, Adelaide, South Australia, Australia

[4] School of Health and Life Science, Glasgow Caledonian University, Glasgow, UK

[5] Institute for Health and Sport, Victoria University, Melbourne, Australia

**Correspondence address:*

Jana Pelclová, PhD

Institute of Active Lifestyle

Faculty of Physical Culture

Palacký University Olomouc

Tř. Míru 117, Olomouc, 771 11, Czech Republic

Phone: +420 585 636 469

Email: jana.pelclova@upol.cz

To allow interpretation of each movement behavior (i.e. sedentary behavior [SB], light physical activity [LPA] and moderate-to-vigorous physical activity [MVPA]) relative to other movement behaviors, the following 3 multivariate multiple regression models were considered:

$\left(z_1^{(MB_\triangle)}, z_2^{(MB_\triangle)}\right) = \boldsymbol{\beta}_0 + \boldsymbol{\beta}_1 Adiposity_1 + \boldsymbol{\beta}_2 Adiposity_\triangle + \boldsymbol{\beta}_3 z_1^{(MB_1)} + \boldsymbol{\beta}_4 z_2^{(MB_1)} + \boldsymbol{\beta}_5 Adiposity_1 + \cdots + \boldsymbol{\varepsilon}$, where

$\left(z_1^{(MB_*)}, z_2^{(MB_*)}\right) = \left(\sqrt{\frac{2}{3}} \ln \frac{SB_*}{\sqrt{LPA_* \times MVPA_*}}, \frac{1}{\sqrt{2}} \ln \frac{LPA_*}{MVPA_*}\right)$ in the first model,

$\left(z_1^{(MB_*)}, z_2^{(MB_*)}\right) = \left(\sqrt{\frac{2}{3}} \ln \frac{LPA_*}{\sqrt{SB_* \times MVPA_*}}, \frac{1}{\sqrt{2}} \ln \frac{SB_*}{MVPA_*}\right)$ in the second model and

$\left(z_1^{(MB_*)}, z_2^{(MB_*)}\right) = \left(\sqrt{\frac{2}{3}} \ln \frac{MVPA_*}{\sqrt{SB_* \times LPA_*}}, \frac{1}{\sqrt{2}} \ln \frac{SB_*}{LPA_*}\right)$ in the third model, with

symbol * stands for either $\triangle$ (i.e. the difference between follow-up and baseline) or 1 (i.e. baseline) and $(SB_\triangle, LPA_\triangle, MVPA_\triangle) = \left(\frac{SB_2}{SB_1}, \frac{LPA_2}{LPA_1}, \frac{MVPA_2}{MVPA_1}\right)$, where 2 stands for follow-up.

That is, as a response we set the difference in the movement composition expressed in pivot coordinates (specific isometric log-ratio coordinates) and as an explanatory variable we set the baseline adiposity indicator, the difference in adiposity indicator, baseline movement composition expressed in pivot coordinates plus the additional covariates. A positive beta suggests that an increase in the respective explanatory variable is associated with an expected increase in the time spent in the movement behavior that is in the numerator of the pivot coordinate at the expense of the movement behavior(s) that is(are) in the denominator of the pivot coordinate. A negative beta suggests that an increase in the respective explanatory variable is associated with an expected decrease in the time spent in the movement behavior that is in the numerator of the pivot coordinate in favor of the movement behavior(s) that is(are) in the denominator of the pivot coordinate.
